# Time trends in proximal humeral fractures from 1944 to 2020 – A cohort study in Malmö, Sweden

**DOI:** 10.1186/s12891-024-07602-y

**Published:** 2024-06-24

**Authors:** Anton Cederwall, Magnus K Karlsson, Björn E Rosengren

**Affiliations:** Clinical and Molecular Osteoporosis Research Unit, Departments of Orthopedics and Clinical Sciences, Skåne University Hospital Malmo and Lund University, Malmö, 205 02 Sweden

**Keywords:** Proximal humeral fracture, Time trends, Epidemiology, Age-adjusted incidence, Seasonal variation

## Abstract

**Background:**

Most studies infer increasing incidence of proximal humeral fractures (PHF) from the 1950´s until the 1990´s. Recent time trends are less clear.

**Objectives:**

Our primary objective was to identify time trends in the age- and sex-adjusted adult incidence of PHF in Malmö, Sweden, from year 1944 until 2020. Our secondary objectives were to describe the variation in incidence according to age, the monthly distribution, and to compare data from the two most recent decades with earlier.

**Study design and methods:**

Malmö has one emergency hospital where acute fractures are treated. We identified PHF in adult patients (≥ 18 years) by reviewing relevant radiology examinations during 17 sample years from year 1944 to 2020. We used jointpoint analyses to estimate time trends.

**Results:**

We identified 3 031 PHF during the study period (3 231 161 person years), 73% were sustained by women with mean age of 69 years (mean age in men 59). Joinpoint analyses indicated an increase in the age- and sex-adjusted incidence of PHF from year 1944 (52 per 100 000 person years) until 1977 (120 per 100 000) and thereafter a decrease until 2020 (85 per 100 000). A seasonal variation with more fractures during winter months, was apparent in earlier but not recent decades.

**Conclusions:**

The age- and sex-adjusted incidence of PHF increased in Malmö, Sweden, from the 1940´s until year 1977 and thereafter decreased until 2020. More fractures were seen during winter months in earlier but not recent decades.

## Background

Proximal humeral fractures (PHF) account for about 10% of all non-spinal fractures in adults [1] with an incidence rate in Sweden of 120 fractures per 100 000 person-years in year 2012 [2]. The mean age in adults sustaining a PHF has been reported to 68 years, with 73% being women [1]. PHF is a potentially devastating fracture in the elderly, associated with mortality and health care costs higher than from distal radial fractures [3].

Studies from Sweden and Finland have identified increasing incidence rates of PHF from the 1950´s until the 1990´s [4, 5]. A previous study from southern Sweden (including the city of Malmö) indicated increasing incidence between year 1999 and 2010 [6] while the incidence in Denmark between 1996 and 2018 [7] and the US between 1999 and 2005 [8] seemed stable. Thus, recent time trends in the incidence of PHF are unclear. Most epidemiological studies agree that the incidence of PHF increases with age, after the age of 50 more pronounced in women than in men [4, 9–11], and that a seasonal variation is evident, at least in northern Europe, with more fractures during winter [9, 11, 12].

Long term epidemiological time trends are important for planning future health care resources, to identify risk factor transitions by period, and patient groups as well as activities where preventive interventions are possible. We have however in the literature only been able to find one long-term epidemiological time trend study, spanning the period 1970–2015, this study however only included female inpatients ≥ 80 years old [13]. There are other studies that include both adult men and women, and in- and outpatients, but these studies span only periods between 2 and 11 years [2, 9, 11, 14].

The primary objective of this study was to describe time trends in the age- and sex-adjusted incidence of PHF in the city of Malmö, Sweden, from year 1944 until 2020 in adults (≥ 18 years old). The secondary objectives were to describe the variation in incidence according to age, the monthly distribution, and to compare data from the two most recent decades with earlier.

## Methods

Malmö is a city in southern Sweden with 131 718 inhabitants in year 1944 and 273 455 in 2020. There is only one hospital where acute fractures are treated.

We reviewed relevant radiology examinations during 17 sample years and recorded information on adult inhabitants (≥ 18 years old) of Malmö who suffered non-pathological fractures of the proximal humerus.

We defined PHF as a fracture within the proximal humerus region, i.e., within the area created by a square with sides equal to the caput humeri width in accordance to the AO Fracture and Dislocation Classification Compendium [15].

From year 1944 to 2003 radiology examinations undertaken at the University Hospital in Malmö were saved in an analogue archive. Records for 1996–2000 were unavailable as they were damaged by a flood. From 2004 and thereafter all new radiology examinations have been saved in a digital archive. The analogue archive is arranged by anatomical region and outcome, i.e., “S.F 1” where S is the region (shoulder), F indicates that the examination displays a fracture, and the reference number is unique for every patient. One of the authors (A.C) reviewed radiographs with the code S.F and brach.F (i.e., a fracture in the shoulder- and brachialis region) and registered all PHF regarding the years 1944–1946, 1952, 1957, 1962, 1967, 1972, 1977, 1981, 1987, 1994 and 1995. Aside from PHF the reviewed codes included fractures in adjacent regions, for example scapula-, clavicle- and diaphyseal humeral fractures. Regarding the years 2005, 2010, 2015 and 2020 we searched the hospital’s in- and outpatient records for the ICD diagnose S42.20, i.e., fracture of the upper end of the humerus and included only habitants of Malmö. One of the authors (A.C) reviewed the digital radiology examinations for identified patients using Sectra IDS7, version 24.1 (Sectra AB, Teknikringen 20 SE-58,330 Linköping, Sweden). Case-finding of fractures by ICD-codes has previously been validated in our setting both in relation to x-ray examinations as well as to review of medical charts [6, 16]).

Official information on the population at risk was retrieved from Statistics Sweden. For years 1944–1967, sex-specific population counts in 1-year age classes were available every fifth year starting at 1945 (except 1955). For sample years without available population details, we used linear regression of the two nearest available years (taking date of collection into account). From 1968 annual sex-specific population figures in 1-year classes were available. The mid-year population regarding the years examined for fracture occurrence was calculated using these population data.

The age- and sex-adjusted incidence rates were calculated using direct standardization with the mean mid-year population during the investigated period, i.e., years 1944–2020, as standard population. We used joinpoint regression analysis (Joinpoint Regression Program, Version 4.9.1.0 - April 2022; National Cancer Institute) for analysis of time trends and present results as annual percent changes (APC) with 95% confidence intervals (CI).

In subgroup analysis we stratified patients by age into two groups, i.e., younger (18–49 years old) or older (≥ 50 years old). The reasons for this cut-off point include the well-established differences in incidence and female/male ratio between these age-groups [17] and to facilitate comparisons to previous studies. We also stratified period into earlier (years 1944–1995) and the two most recent decades (2005–2020). The reason for this cut-off point was to identify any recent trend changes, which would be useful for estimating future health care burden.

For analyses on monthly distribution, we calculated the month- and year-group specific incidence rates with year 1977 as reference. We used analysis of variance (ANOVA) to assess the variance, during years 1944–2020 (in the full dataset of 17 sample years) between months (January to December) and during 1944–1995 and 2005–2020 (subgroups) between month-groups (January + December compared to February-November). We tested differences between pairs of months by Tukey’s test. P-values < 0.05 were considered statistically significant.

We used SPSS v28.0 (IBM SPSS Statistics for Macintosh, Version 28.0. Armonk, NY: IBM Corp) and Microsoft Excel v16.67 (Microsoft Corp., Redmond, WA, USA) for database management and statistical analysis.

### Ethical approval

of the study was obtained prior to study start from the regional ethical review board of Lund University. The need for informed consent was waived by the regional ethical review board of Lund University (LU 2012 − 394).

## Results

We reviewed a total of 6 064 radiology examinations and identified 3 031 PHF during the 17 sample years (3 231 161 person years), ranging from 42 PHF in 1944 to 232 PHF in 2020. The overall incidence rate was 94 per 100 000 person years (130 in women and 53 in men). 73% of all fractures affected women. The overall mean age at the time of fracture was 67 (Standard deviation (SD) 16) years, 69 (SD 14) in women and 59 (SD 17) in men. 52.5% of all fractures affected the left side (53.1% in women, 50.9% in men) (Table 1).

**Table 1:** Sex-, age- and fractured side distribution in proximal humeral fracture patients, Malmö, Sweden 1944–2020

**Sex (** ***n*** ** = 3031)**			***n*** **(%)**
	Male		815 (27%)
	Female		2216 (73%)
**Age (** ***n*** ** = 3031)**			**mean year (SD)**
	Total		66.8 (15.8)
	Men		59.5 (17.2)
	Women		69.5 (14.3)
**Fractured side (** ***n*** ** = 3016)**			**n (%)**
	Total	Right	1433 (48%)
		Left	1583 (52%)
	Men	Right	399 (49%)
		Left	413 (51%)
	Women	Right	1034 (47%)
		Left	1170 (53%)

### Time trends in age adjusted incidence

**Fig. 1:**
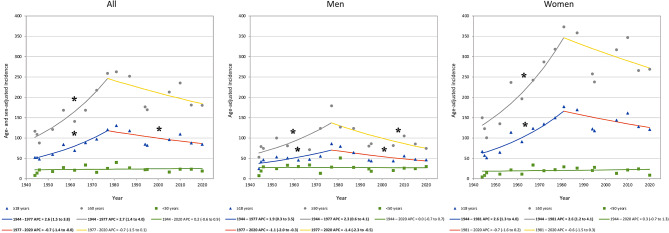
Time trends in the incidence of proximal humeral fractures. Sex- and age-group-specific time trends in the age- and sex-adjusted incidence of proximal humeral fracture in Malmö, Sweden, 1944–2020 presented as annual percent change (APC) with 95% confidence interval (95% CI). For clarity, statistically significant changes are marked by * with bolded legend

**Fig. 2:**
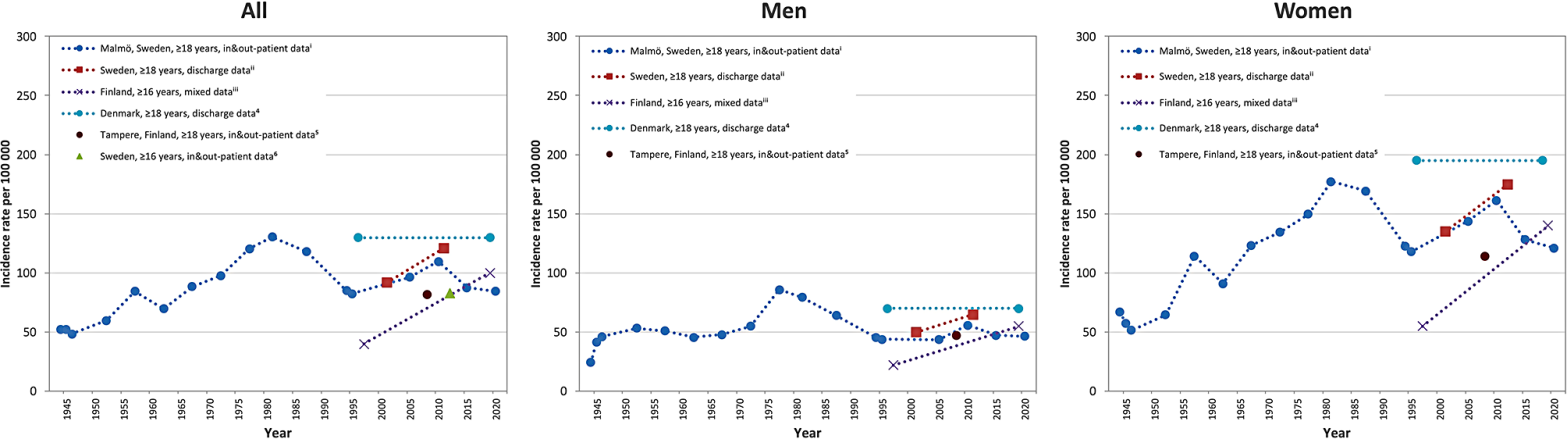
Sex-specific incidence of proximal humeral fractures in different settings and time periods. Sex-specific incidence per 100 000 of proximal humeral fracture in different settings and time periods. ^i^ Current study. ^ii^ Sumrein et al. 2017. ^iii^ Leino et al. 2022. 1997–2011 discharge data. 2011–2019 in&out patient data. ^4^ Brorson et al. 2022. ^5^ Launonen et al. 2015. ^6^ Bergdahl et al. 2016

Joinpoint analyses indicated that the age- and sex-adjusted incidence of PHF increased from the 1940`s until 1977 (APC + 2.6% [95% CI + 1.5 to + 3.8]) followed by a decrease until 2020 (APC − 0.7% [95% CI -1.4 to -0.0]) (Fig. 1). In sex specific analyses, the age-adjusted incidence of PHF increased in men from 1944 to 1977 (APC + 1.9% [95% CI + 0.3 to + 3.5] and in women from 1944 to 1981 (APC + 2.6% [95% CI + 1.3 to + 4.0]. After 1977 we found a decrease in men (APC − 1.1% [95% CI -2.0 to -0.3]) while the decrease in women that we saw from 1981 to 2020 did not reach statistical significance (APC − 0.7% [95% CI -1.6 to + 0.2]) (Fig. 1). Similar time trends were seen in sub-group analysis of patients ≥ 50 years old, while no time trends were apparent in age group 18–49 years (Fig. 1). The sex-specific PHF age-adjusted incidence rate in the present study in relation to other, comparable, studies is presented in Fig. 2.

### Incidence variation in relation to sex and age

**Fig. 3:**
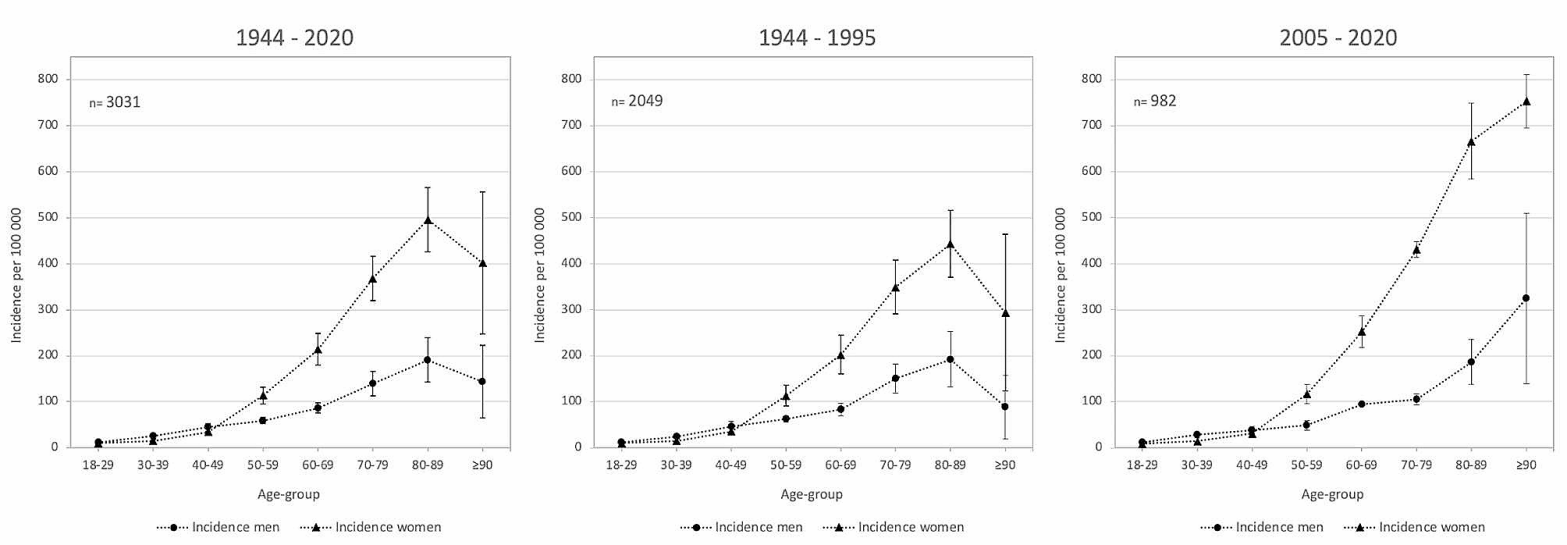
Sex-specific incidence of proximal humeral fractures in 10-year age groups. Sex-specific sample year-average incidence per 100 000 of proximal humeral fracture in 10-year age groups in Malmö, Sweden years 1944–2020, 1944–1995 and 2005–2020. Error bars represent 95% confidence intervals

In age-groups 18–49 years we found similar incidence of PHF in men and women. In age-groups ≥ 50 years old the incidence of PHF was higher in women than men. This pattern was seen in both distant (years 1944–1995) and recent (2005–2020) decades (Fig. 3).

### Monthly distribution

**Fig. 4:**
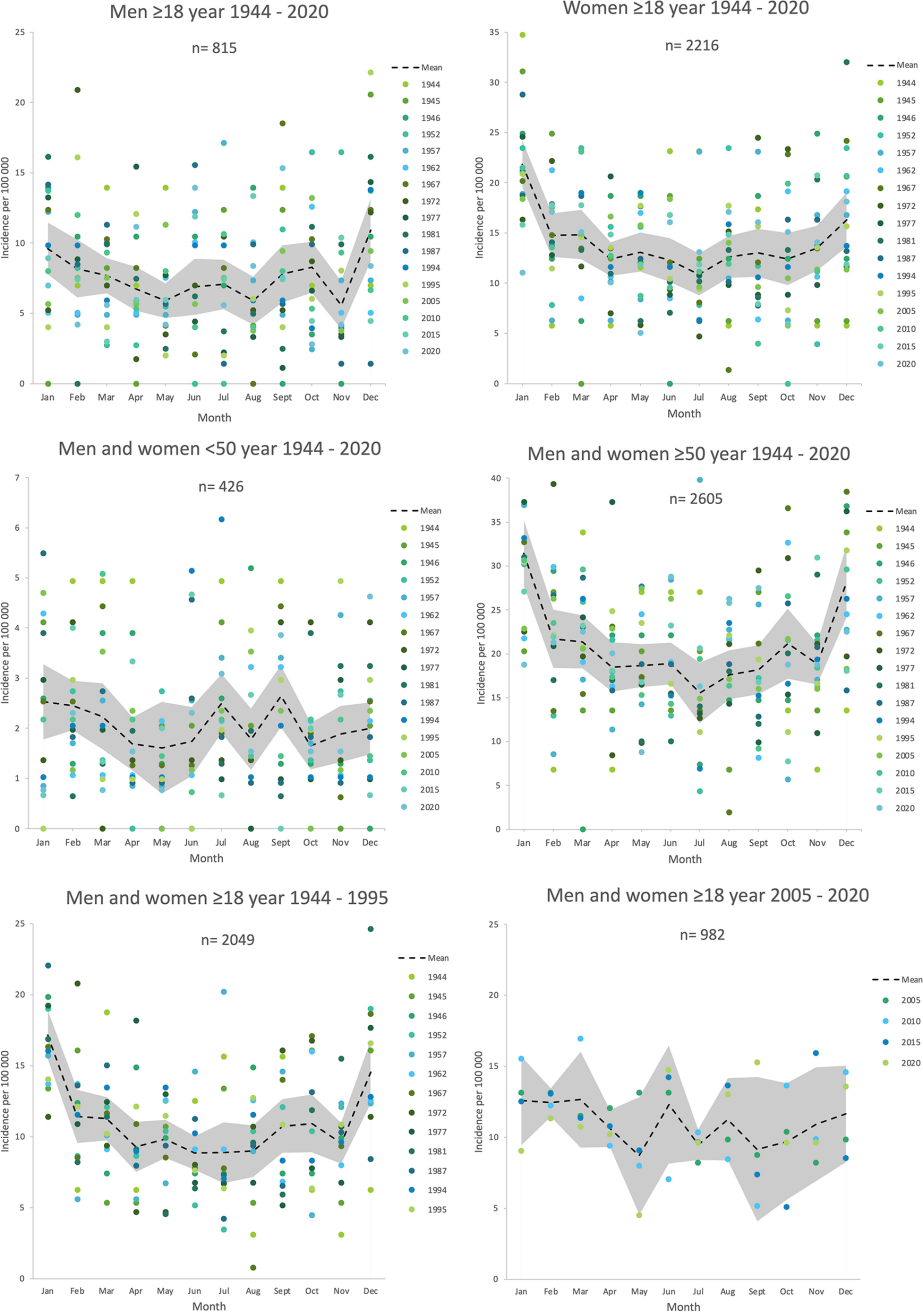
Monthly distribution of proximal humeral fracture. Year-weighted sex-, age- and year-group-specific monthly distribution of proximal humeral fracture incidence in Malmö, Sweden during 17 sample years 1944–2020. Shaded areas represent 95% confidence intervals and colored dots individual year data

We found a seasonal variation, with higher incidence of PHF in the colder winter months, in all patients ≥ 50 years old and in women 1944–2020, as well as in all patients during distant decades (1944–1995) (ANOVA, *p* < 0.001). We were not able to identify seasonal variation in all patients < 50 years old 1944–2020 (ANOVA, *p* = 0.408) and in all patients during recent decades (2005–2020) (ANOVA, *p* = 0.228) (Fig. 4; Table 2). Variance test details, i.e., variances between months, are presented in Table 2.

**Table 2:** Variance of mean proximal humeral fracture incidence between months

	Variance^a^	Variance between months^b^		
	*p*-value	Peak month^c^	Lower month(s)^d^	*p*-value
**All**	< 0.001	Jan	All except Dec	All ≤ 0.037
**Men**	0.022	Dec	Nov	≤ 0.030
**Women**	< 0.001	Jan	All except Dec	All ≤ 0.017
**≥ 50 year**	< 0.001	Jan	All except Dec	All ≤ 0.020
**< 50 year**	0.408	Sept	n/a	n/a
**1944-1995** ^**e**^	< 0.001			
**2005-2020** ^**e**^	0.228			

^a^ ANOVA. ^b^ Tukey´s test. ^c^ Month with highest mean incidence. ^d^ Month(s) with statistically significantly lower incidence than peak month.

^e^ Variance between month-groups (Jan + Dec compared to Feb-Nov).

Sex-, age-group- and year-group-specific monthly variance of mean proximal humeral fracture incidence and variance between months in Malmö, Sweden 1944–2020.

## Discussion

The age- and sex-adjusted incidence of PHF increased in Malmö, Sweden, from the 1940´s until 1977 and thereafter decreased until 2020 (Fig. 1). The incidence was similar in men and women in age group 18–49 years and higher in women than men in age group ≥ 50 years (Fig. 3). A seasonal variation, with more fractures during winter months, was apparent in earlier but not in recent decades (Fig. 4; Table 2).

Our finding of an increasing incidence from the 1940´s until 1977 is in line with prior studies [4, 5]. Several possible explanations to this have been proposed; e.g., a more fragile skeleton, partly mediated through lower level of physical activity and changes in life style or a shift towards activities with a higher risk of trauma [4].

Palvanen et al. described an increasing PHF incidence, patients ≥ 60 years old, in Finland between 1970 and 2002. When we reviewed their incidence figures a possible trend change, at least in women, may have occurred about year 1995 with a following levelling off until 2002 [5]. This is in analogy with another study from Finland, patients ≥ 80 years, which however only included in-patients [13]. The trend change in our study was seen at the end of the 1970´s. The incidence curves resemble each other, with the difference that the trend change in our study occurred almost two decades earlier. The reasons for the trend change are unknown. Possible explanations include improved functional ability in the elderly, effects of preventive programs and improved bone strength (less smoking, vitamin D, calcium, bone-specific agents and hormone replacement therapy (HRT)). It is possible that changes in these and other fracture risk modulating factors occurred earlier in Sweden than Finland or were different in some way resulting in different times for trend changes.

HRT was widely prescribed to postmenopausal women during the 1980 and 1990´s [18]. A metanalysis described a 27% reduction of overall clinical, non-vertebral, fractures in women with at least 12 months of HRT treatment compared with placebo [19]. In women, the HRT effect on fracture risk could possibly contribute to the decrease in incidence seen during the early 1990´s (Fig. 1). During the 1990´s reports on adverse effects of HRT came into light leading to a decrease in HRT prescription and usage [20]. We could not see any major subsequent increase in incidence thereafter, making it reasonable to assume that other risk factors also affected the PHF risk. It is probable that bone resorption agents have contributed to a lower PHF occurrence in the population. In Sweden these were however introduced in the late 1990s, with low initial prescription rates making other responsible factors more probable for the found trend changes in late 1970s/ early 1980s.

Another contributor to the decreasing incidence during the 1990´s and onwards may be immigration. Albin et al. reported a reduced hip fracture risk in foreign- compared to Swedish-born persons living in Sweden between year 1987 and 1999 based on national data [21]. During the 1980´s there was an increasing immigration to Sweden and Malmö, especially from Iran, Chile, Lebanon, and Turkey and in the early 1990´s also immigrants from former Yugoslavia [22]. Fracture incidence rates in these countries are generally lower than in Sweden [23] which may have contributed to the decreasing PHF incidence seen during the 1990´s in our data.

Brorson et al. reported a stable incidence in Denmark between year 1996 and 2018 using both in- and outpatient data [7]. This trend is similar to our figures during the same years (Fig. 1). Brorson et al. and Sumrein et al. reported an incidence rate of about 120 in 2010 while we found 110 per 100 000. The different case-finding strategies, where we confirm all fractures by reviewing radiographs, may in part explain the lower incidence rate in the present study. Another contributing factor may be that the population in Malmö is more multi-ethnical, as incidence rates of PHF seem to vary with origin, as mentioned previously.

Sex specific analyses revealed that the incidence for men increased from year 1944 to 1977 followed by a decline until 2020. For women, incidence increased between 1944 and 1981 whereafter a trend for decrease was seen, but this did not reach statistical significance, APC − 0.7% (95% CI -1.6 to + 0.2). The same patterns were seen in sub-group analysis of older patients (≥ 50 years old). In sex-specific analysis in younger individuals (18–49 years old) incidence rates seemed stable from 1944 to 2020 in both women and men (Fig. 1). To our knowledge, long-term time trend sub-group analysis of the incidence of PHF like this have not previously been reported.

Bergdahl et al. reported on humeral fractures between year 2011 and 2013 in patients ≥16 year in Gothenburg, Sweden. They described a higher incidence of PHF in older age groups in both men and women [9] which corresponds well with our results in recent decades (2005–2020) (Fig. 3). In distant decades (1944–1995) we found a similar incidence increase by age except in the oldest age-group (≥90 years old) where the incidence was lower than the adjacent younger age group. The reasons behind this are unknown but age-group ≥90 years may contain a higher proportion of fragile patients in recent than distant decades. We, like reports from Germany 2007–2016 [17] and Sweden 2011–2013 [9], found similar incidence rates in women and men in age-group 18–49 and higher in women than men in age-group ≥50 years. This pattern emerged also in sub-group analyses of recent and earlier decades. This suggests that the PHF population can be considered as two different entities, i.e., patients 18–49 years old and ≥50 years of age. In patients 18–49 years old the incidence is low and similar in men and women. In patients ≥50 years old the incidence is high with a female dominance, probably attributable to risk factors mainly affecting women, e.g., menopause, lower estrogen levels and a more fragile skeleton.

We found a seasonal variation, with higher incidence during the colder winter months. This has previously been described in Sweden, Finland, and the United Kingdom [9, 11, 12]. The seasonal variation was apparent in women, in patients ≥50 years old and during distant decades (years 1944–1995) but not in patients 18–49 years old or during recent decades (2005–2020). To our knowledge, these observations have not been seen previously and need to be verified by future studies. Several factors probably contribute to the change but due to the design of the study we can only speculate on these. The mean temperature in Sweden has increased from the 1980´s until 2016 [24] as has the number of days without snow in Southern Sweden [25]. This may have resulted in less slippery weather conditions in recent decades as well as lower participation in certain winter activities that may be associated to higher fracture risk, e.g., ice skating or skiing. Better and more widely used spiked shoes and more effectively salted walkways/roads may also have contributed. Fracture occurrence in Rome, Italy [14], with less icy weather conditions than in southern Sweden, describes a seasonal variation regarding PHF but seemingly not as strong as the figures from Finland, Sweden [9, 11] and in parts of the present study.

We obtained official population data from Statistics Sweden. These were however from two different time periods, with some differences which may be regarded as a limitation. From 1940´s until year 1967 data were only available for certain years. To estimate population figures for actual years of fracture collection where data were unavailable, we extrapolated population numbers from available years. Regarding 1968 and onwards, populations data were available every year. The definition of Malmö was somewhat different for the timed periods, i.e., “Malmö and its suburbs” earlier and “Malmö municipality” later which however are largely corresponding.

Another limitation is that we were unable to verify that every patient in the old archive (years 1944–1995) was a resident of Malmö. This means that some patients residing outside Malmö, that for some reason sought health care for PHF at the hospital in Malmö, may have been included. Malmö residents who suffered PHF outside Malmö have probably been included as follow up examinations in Sweden usually take place at the home hospital. Regarding year 1981, where more detailed data of the patients were available, we found that only 3 of 288 (1.0%) patients were habitants elsewhere. Giving that the rest of the examined years probably have a similar distribution, the overall effect seems very limited, yet a small overestimation of the incidence during early years may thus be possible. Regarding the new archive (2005–2020) data on the patient’s residence were available, and only patient’s residing in Malmö were included.

Other aspects that may have influenced our result is that patients could have sought health care more seldom and may have been radiographically examined to a lesser extent in distant than recent years. These factors are speculative but could possibly contribute somewhat to the apparent increase in incidence from 1940´s to the late 1970’s or early 1980´s.

Plain radiographs are often considered gold standard in fracture ascertainment, but archives are seldom validated. In the analogue archive we found patients from the beginning of January until the end of December every year and no or very few skipped reference numbers. We are however unable to rule out that some odd fractures may have been missed. A review of radiology examinations from every year from 1944 until 2020 would have been optimal. Our examination of 17 sample years distributed throughout the study period was a more feasible alternative which still enabled examination of trends and patterns.

Our results from Malmö are probably not immediately generalizable to substantially different settings such as developing countries, the mixed population in the United States or to Asian megacities. However, Sweden has one of the highest reported fracture incidences in the world [26] which together with well-validated official records enable detection of emerging trends.

A major strength of this study is the case finding strategy with review of radiology examinations. This means that only objectively verified fracture cases are included, in contrast to most other studies that rely solely on register data [2, 5, 7, 9, 13, 27–29]. This method also enables inclusion of in- as well as outpatients, in contrast to studies that only include inpatients [5, 13, 30]. The study period of 80 years should also be considered a strength and facilitates detection of relevant major time trends during a relatively long period.

## Conclusion

The age- and sex-adjusted incidence of PHF increased from the 1940´s until year 1977 in Malmö, Sweden, and thereafter decreased until 2020. Seasonal variation, with more fractures during winter months, was apparent earlier but not in recent decades (2005–2020). The reasons for the changes are unknown and important to examine further as is time trends in fracture severity.

## Data Availability

The dataset used during the current study are available from the corresponding author on reasonable request.

## Abbreviations

PHF: Proximal humeral fracture
APC: Annual percent change
CI: Confidence interval
ANOVA: Analysis of variance
SD: Standard deviation
HRT: Hormone replacement therapy
